# Dry Amorphization of Itraconazole Using Mesoporous Silica and Twin-Screw Technology

**DOI:** 10.3390/pharmaceutics16111368

**Published:** 2024-10-25

**Authors:** Margarethe Richter, Simon Welzmiller, Fred Monsuur, Annika R. Völp, Joachim Quadflieg

**Affiliations:** 1 Thermo Fisher Scientific, Pfannkuchstr. 10-12, 76185 Karlsruhe, Germany; annika.voelp@thermofisher.com; 2 Thermo Fisher Scientific, Im Steingrund 4-6, 63303 Dreieich, Germany; simon.welzmiller@thermofisher.com; 3 Sil’innov, Rue de Liege 2, 6180 Courcelles, Belgium; f.monsuur@silinnov.eu; 4 Grace GmbH, In der Hollerhecke 1, 67547 Worms, Germany; joachim.quadflieg@grace.com

**Keywords:** twin-screw granulation, twin-screw extruder, API amorphization, solubility enhancement, mesoporous silica, continuous manufacturing

## Abstract

**Background/Objectives:** Amorphization of an active pharmaceutical ingredient (API) can improve its dissolution and enhance bioavailability. Avoiding solvents for drug amorphization is beneficial due to environmental issues and potential solvent residues in the final product. **Methods:** Dry amorphization using a twin-screw extruder is presented in this paper. A blend of mesoporous silica particles and crystalline itraconazole was processed using a pharma-grade laboratory scale twin-screw extruder. The influence of different screw configurations and process parameters was tested. Particle size and shape are compared in scanning electron microscopy (SEM) images. Differential scanning calorimetry (DSC) and X-ray diffraction (XRD) are used to determine the residual amount of crystalline itraconazole in the final product. **Results:** An optimized screw configuration for the process was found which leads to more than 90% amorphous API when processed at room temperature. Full amorphization was reached at 70 °C. The specific mechanic energy (*SME*) introduced into the material during twin-screw processing is crucial for the dry amorphization. The higher the *SME*, the lower the residual amount of crystalline API. Two months after processing, however, recrystallization was observed by XRD. **Conclusions:** Dry processing using a twin-screw extruder is continuous, free of solvents and can be performed at low temperatures. This study proves the concept of twin-screw processing with mesoporous silica for dry amorphization of itraconazole.

## 1. Introduction 

The oral administration of drugs requires solubility in the gastrointestinal fluid before its absorption into the bloodstream. However, 90% of new chemical entities and 40% of the top solid oral drugs marketed in the U.S. and Europe are poorly water soluble [1,2]. Generally, crystalline APIs possess high purity and physio-chemical stability, but the crystal lattice energy barrier hinders their dissolution. This results in the low bioavailability of the active pharmaceutical ingredient (API), as it is limited by its solubility and permeability. An API in its amorphous state inheres increased solubility but is thermodynamically unstable and recrystallisation needs to be prevented by excipients or carriers [3]. Typically, these are polymers, e.g., polyvinyl pyrrolidone (PVP) or hydroxypropyl methylcellulose (HPMC) [4]. Recently, inorganic mesoporous carriers possessing high numbers of nano-scale pores, have shown improved stability of the amorphous state of some APIs [5,6,7,8,9,10]. Amorphization can either be achieved by the dissolution of the API crystals in a solvent or by solvent-free methods, i.e., melting or milling [11]. Milling bears the advantages of a solvent-free process with preferentially low temperature impact on the API. The high internal surface area in combination with the intermolecular hydrogen bonding between the API and the carrier results in a stable amorphous single-phase system [5,12]. Solvent-free amorphization of APIs with mesoporous silica in a batchwise ball milling process has shown to improve the API’s dissolution performance [8,12,13,14,15]. However, this processing route requires long milling times (15 min or more) [8,13]. Transferring this batch process into continuous manufacturing promises a reduction in production down-time and costs, faster development times, increased reproducibility, and flexibility of production [16,17]. Twin-screw extruders are used for continuous granulation with controlled throughput, specific mechanical energy introduction, and material temperature. Extruders in twin-screw granulation (TSG) mode allow for the wet and dry processing of pharmaceutical ingredients. In this study, a solvent-free dry granulation process is evaluated for the amorphization of crystalline itraconazole with mesoporous silica powder. The factors with the biggest impact on the process are expected to be the configuration of the screws, the residence time during twin-screw processing, and the specific mechanical energy introduced into the material. However, to our knowledge, this has not been analyzed in detail yet. Therefore, the focus of this study is to evaluate different screw configurations and process settings and their influence in the residual crystallinity of the API in the formulation.

## 2. Materials and Methods

### 2.1. Materials

Mesoporous silica powder (SYLOID XDP 3050 E551, Grace, Worms, Germany) and the API itraconazole (Sporanox, Janssen Pharmaceutica, Beerse, Belgium) mixed in equal weight fractions (weight fraction itraconazole ω_i_ = 0.5). The blending was performed by manual mixing of a 500 g batch in a container of 2 L size. To homogenize the blend, it was pushed through a sieve with a mesh size of 710 µm.

### 2.2. Twin-Screw Processing

The pre-blend was fed by a gravimetric dosing system (gravimetric MiniTwin, Kubota Brabender Technologie, Duisburg, Germany) into a lab-scale twin-screw extruder (Thermo Scientific^TM^ Pharma 11 Twin-screw extruder, Thermo Fisher Scientific, Karlsruhe, Germany). The extruder was equipped with a twin-screw granulation (TSG) kit. This includes granulation screws of 11 mm diameter and 448.25 mm length and an open discharge chute instead of a die. For a picture of the set-up, see Supplementary Information Figure S1.

**Figure 1:**
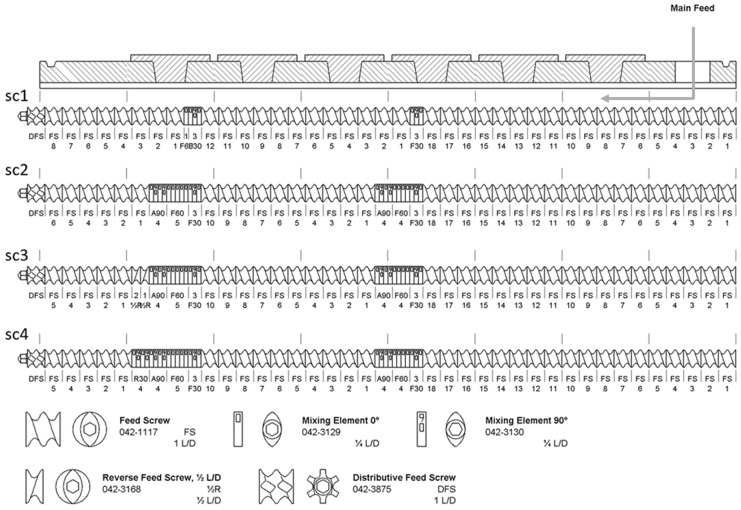
Screw configurations sc1 to sc4 for the Pharma 11 extruder in TSG mode.

Four different screw configurations were tested, each containing two kneading zones with different lengths and different offset angles between the mixing elements, see Figure 1. The change in the screw configuration is based on analysis data (drawn from DSC, see Section 2.3). The screw speed was varied between 30 rpm, 100 rpm, and 250 rpm. The feeding rate of the blend was 0.1 kg/h or 0.5 kg/h. Different temperature settings were tested as follows: room temperature (25 °C), 70 °C, and temperatures above the melting point of itraconazole (175 °C and 200 °C). The mean residence time (MRT) in the twin-screw process was estimated manually based on the maximum intensity of the tracer (iron black) at the outlet of the extruder. The specific mechanical energy (*SME*) introduced into the material by the twin screws is determined by

(1) $$SME=\frac{2\Pi n\tau }{\;\dot{M}}$$

With screw speed n, extruder torque τ, and throughput $\dot{M}$.

### 2.3. Differential Scanning Calorimetry (DSC)

First, the analysis of the raw materials and the resulting powders was performed using differential scanning calorimetry (DSC 204F1 Pheonix (240-12-0023-L), Netzsch, Selb, Germany). An amount of 10 mg of the sample was weighed in an aluminum crucible. The crucible was sealed with a perforated lid. The DSC temperature profile contains heating from 25 °C to 200 °C and cooldown to 25 °C at heating/cooling rates of 10 K/min. The crystalline fraction c of itraconazole (index *i*) in the processed material (index *s*) is calculated via

(2) $$c=\frac{\Delta H_{f,s}}{\Delta H_{f,i}\;\cdot \;\omega_{i}}$$

$\Delta H_{f,s}$ represents the respective heat of fusion of the sample, $\Delta H_{f,i}\;$ the heat of fusion of itraconazole, and *ω_i_* the weight fraction of itraconazole in the sample. Heat of fusion was determined from the DSC signal (area under the melting peak).

DSC is used as a fast analysis tool to improve the process. Based on the results, the screw configuration for twin-screw processing is adapted from sc1 (starting point) to sc4. Further analysis is only performed for the optimized screw configuration. If not directly analyzed after processing, the samples were stored at room temperature in airtight, sealed containers for one month.

### 2.4. Scanning Electron Microscopy (SEM)

The resulting powder from sc4 processing was placed on a standard SEM stub using a double-sided adhesive carbon tape. For the visualization of the samples, a Thermo Scientific™ Apreo S Scanning Electron Microscope (SEM) by Thermo Fisher Scientific (Eindhoven, The Netherlands) was used at high vacuum and 500 V and a T1 detector.

### 2.5. X-Ray Diffractometry (XRD)

The phase composition of the product from processing with screw configuration sc4 was investigated using a powder X-ray diffractometer (Thermo Scientific™ ARL EQUINOX 100, Thermo Fisher Scientific, Germany) with Cu Kα radiation at 36 W power irradiating the sample for 5 min and detecting the reflection with a count per second (CPS) detector. To quantify the amorphous content, the PONKCS method was used [18]. A 1:1 mixture of itraconazole and Syloid was used to set up calibrated peaks. A whole pattern Rietveld refinement was conducted to quantify the amorphous API fraction using Profex software, version 5.3.0 [19]. XRD analysis was performed two months after processing. The samples were stored at room temperature in airtight, sealed containers during this time.

## 3. Results and Discussion

### 3.1. Analysis of Raw Materials

The raw materials and the pre-blend were characterized before the twin-screw process. Pure itraconazole obtains a melting peak in the heating ramp at an onset temperature of 168 °C and an intensity of 289.8 µVs/mg, leading to a heat of fusion $\Delta H_{f,i}\;$= 88 J/g. No recrystallization peak can be observed in the cooling segment during DSC. This effect is known as the subcooling of itraconazole. Similar results can be seen in the DSC of the blend of itraconazole with MPS with a reduction in the melting temperature (onset at 165 °C). MPS has no significant signals in DSC in this temperature range.

### 3.2. Twin-Screw Processing with Screw Configuration sc1

**Figure 2:**
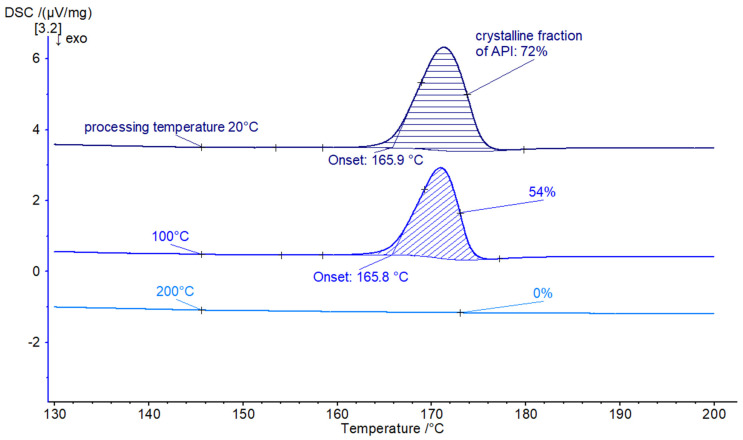
DSC results for processed material with screw configuration sc1, at a screw speed of 30 rpm, a throughput of 0.1 kg/h, and different processing temperatures (20, 100, and 200 °C).

Screw configuration sc1 has two short kneading sections with F30 (forward 30°) mixing, resulting in the mildest twin-screw processing of the material in this study. Roughly 28% amorphization could be observed when processing the material with sc1 at room temperature. An increase in temperature leads to an increase in amorphization (see Figure 2), resulting in a complete amorphization at a processing temperature of 200 °C.

### 3.3. Twin-Screw Processing with Screw Configuration sc2

**Figure 3:**
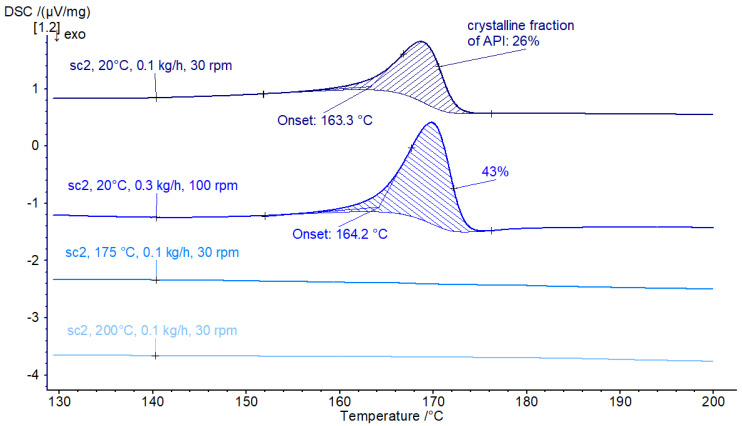
DSC results for processed material with screw configuration sc2.

Increasing the intensity of kneading to the screw configuration sc2 leads to more efficient amorphization at room temperature. For the same process conditions, more itraconazole is brought to an amorphous state. As Figure 3 shows, residual crystallinity is 26% vs. 72% with sc1. Increasing throughput and screw speed leads to an increase in the resulting crystalline fraction of API to 43%. For elevated processing temperatures of 175 °C and above, full amorphization is reached.

### 3.4. Twin-Screw Processing with Screw Configuration sc3

In the screw configuration sc3, the intensity of mixing is increased even further by adding reverse conveying elements behind the second kneading zone. However, with this configuration, the process did not reach a steady state, as the dry powder became blocked in the second kneading zone, leading to an increase in temperature and over-torque in the extruder. Therefore, sc3 cannot be recommended for the dry amorphization process.

### 3.5. Twin-Screw Processing with Screw Configuration sc4

**Figure 4:**
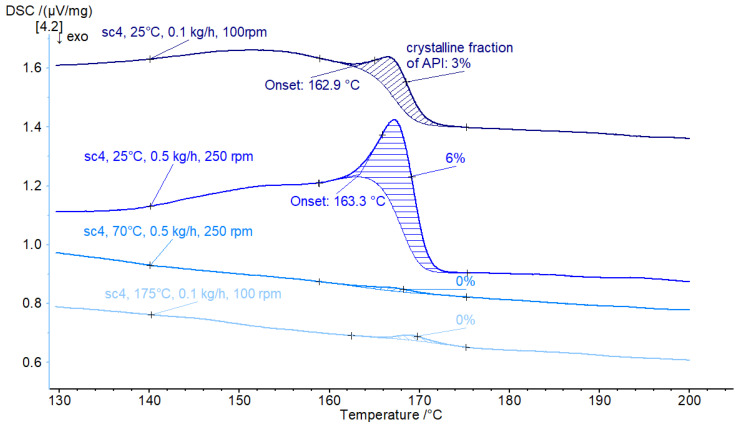
DSC results for processed material with screw configuration sc4. DSC measurement 1 month after processing.

For the screw configuration sc4, the reverse elements in sc3 were replaced by a reverse mixing section (R30). The material could be processed smoothly at two different throughputs (0.1 kg/h and 0.5 kg/h), leading to a residual crystalline fraction of API of 3% and 6%, respectively, see Figure 4. For elevated temperatures (70 °C and 200 °C) full amorphization was observed. However, these DSC measurements were performed 1 month after the twin-screw processing of the samples. A small melting peak shows <0.3% residual crystalline fraction of API, which indicates recrystallization over time.

**Figure 5:**
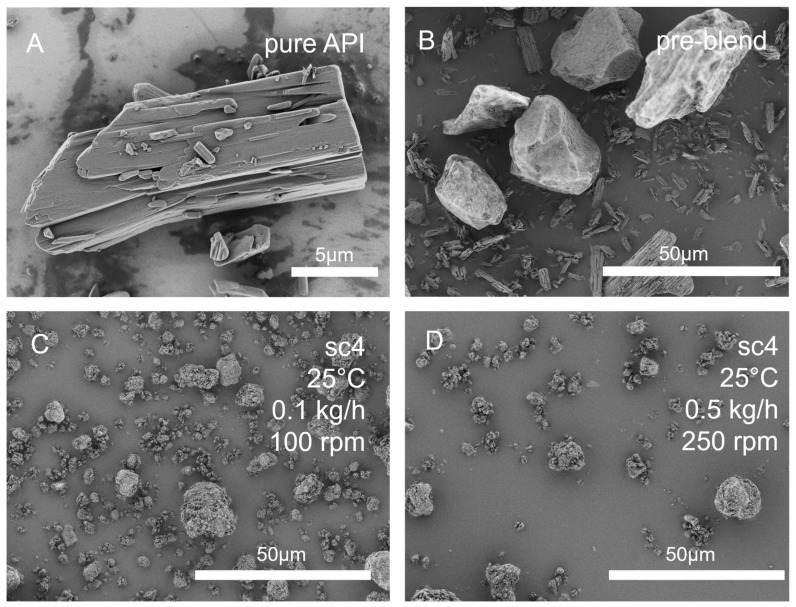
SEM images of (**A**) pure itraconazole, (**B**) pre-blend of mesoporous silica and itraconazole, granules produced by twin-screw granulation at (**C**) 25 °C, 100 rpm screw speed and 0.1 kg/h throughput, and (**D**) at 25 °C, 250 rpm screw speed and 0.5 kg/h throughput. Unprocessed SEM pictures can be found in the Supplementary Figure S2A–D.

Figure 5 displays SEM images of the pure API itraconazole, a mixture of mesoporous silica powder and the API prior to granulation, and the mixture after granulation at various process conditions. The image of the initial mixture shows silica grains several tenths of µm in size and smaller needle-shaped particles, identified as crystalline itraconazole in the SEM image of the pure API. After the granulation process, the mixture exhibits a decrease in maximum grain size down to 10 µm and the absence of any needle-shaped particles on the SEM image, regardless of the process conditions.

The bimodal particle size distribution of the pre-blend is narrowed down, and the average particle size is successfully reduced during the granulation process due to the milling effect of the co-rotating screws. Additionally, the crystalline structure of the API appears to vanish even at process temperatures down to room temperature.

**Figure 6:**
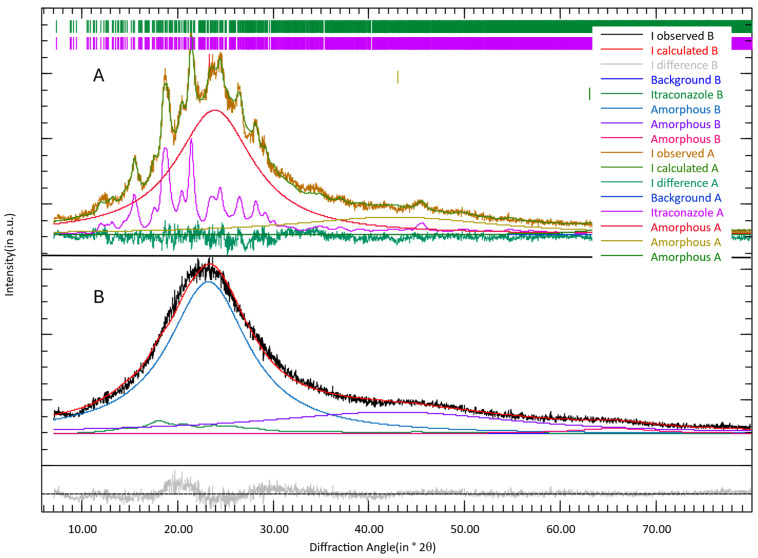
XRD patterns of samples of mesoporous silica and itraconazole produced by twin-screw granulation at (**A**) 25 °C, 250 rpm screw speed, and 0.5 kg/h throughput, and (**B**) 70 °C, 250 rpm screw speed, and 0.5 kg/h throughput.

Figure 6 shows the XRD patterns of the granulate processed with sc4 at 25 °C (A) and 70 °C (B) with 0.5 kg/h throughput. In pattern A (25 °C), reflections corresponding to crystalline itraconazole are visible (in pink). The refinement yields 60 wt% amorphous content and 40 wt% crystalline itraconazole (80 wt% of the initial itraconazole). A crystallite size of 15 nm is determined from the diffraction peak broadening [20] with a strong indication of a broad size distribution [19]. Pattern B (70°) exhibits no distinct reflections, but the broad peak at ~22° 2θ cannot be explained by an amorphous phase only. Therefore, itraconazole was added to the refinement, which significantly increases the statistical parameters. The itraconazole has a very small crystallite size of 4 nm with an indication of a broad size distribution and constitutes 14 wt% of the sample (28% of the initial itraconazole) with 86 wt% of amorphous content. The XRD analysis of the pure mesoporous silica (Syloid) confirmed a complete amorphous structure. Determined by XRD 2 months after processing, the residual crystallinity of the API is 80% when processed at 25 °C and 0.5 kg/h throughput, and 44% when processed at 25 °C and 0.1 kg/h throughput. In the sample produced at 70 °C, only API with a very low crystallite size was traced.

**Figure 7:**
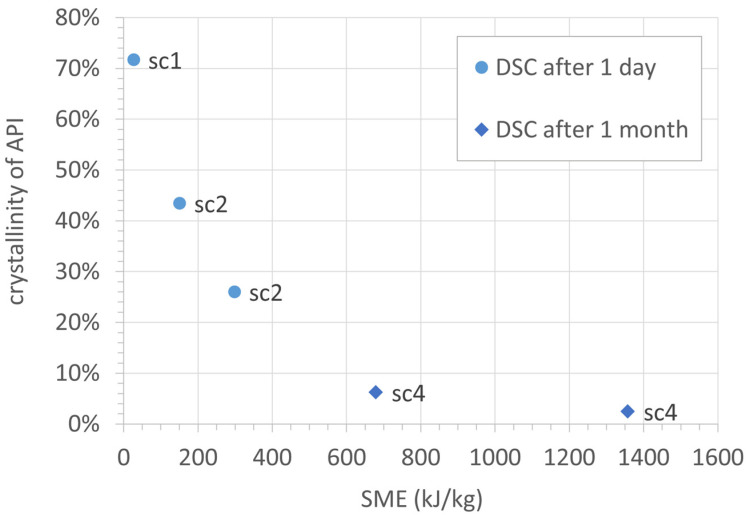
Crystallinity of API over the specific mechanical energy introduced during the twin-screw process with different screw configurations at room temperature.

The XRD analysis confirms the results of the DSC that a fraction of API crystals experience amorphization at 25 °C and crystals entirely vanish at 70 °C during the granulation process. However, a higher crystalline fraction of the API is determined by XRD than by DSC. DSC measurement was performed 1 month after processing the materials, whereas XRD was performed 2 months after processing. Again, this indicates that recrystallization occurs over time. The small crystallite size in the XRD analysis of B (Figure 7) could already be an indication of the starting recrystallization process which follows divergent kinetics compared to the other samples. For a 1:1 blend of API and MPS, traces of residual crystalline API can be expected, leading to seeds for recrystallization. A more detailed study of the kinetics of recrystallization is required, but this was not in the scope of the current study.

### 3.6. Results Summary and Process Discussion

Table 1 summarizes the process data and the results drawn from DSC and XRD. The screw configuration 4 shows the best amorphization behavior at room temperature. XRD data determined 2 months after processing altogether show higher values for the crystallinity of the API. Interestingly, an increase in processing temperature does not necessarily lead to full and stable amorphization after 1 month. Also, the processing conditions as mean residence time and specific mechanical energy (*SME*), introduced into the material, need to be sufficient to reach a fully amorphous product.

Based on the data collected for the different screw configurations and process settings, a dependency of API crystallinity on the specific mechanic energy (*SME*) introduced into the material can be seen. Figure 7 shows a graph summarizing all DSC data collected for samples processed at room temperature. The residual crystallinity after the twin-screw granulation process clearly decreases with an increasing *SME*.

Standard hot-melt extrusion is lubricated by the material. In the dry amorphization process shown here, there is no lubrication. Therefore, there might be the risk of abrasion of the process parts. During the twin-screw processing of the blend, no color change in the powder could be observed, and the surface of the barrel and the screw did not show any marks. In addition, XRD measurements did not detect any traces of iron or other metals in the samples (see Supplementary Data Figure S3).

## 4. Conclusions

The granulation of a blend of mesoporous silica particles and the API itraconazole in a twin-screw extruder reduced the particle size, as well as the amount of crystalline API, as derived from SEM imaging. DSC analysis evidenced complete amorphization of the API at 70 °C, well below the glass transition temperature. At room temperature, over 90% of the API was amorphous after granulation. The dependency of residual API crystallinity and the specific mechanical energy introduced into the material during the process was found. The more specific mechanical energy is introduced into the material the higher the amorphization fraction of the API. However, recrystallization after 2 months could be observed by the XRD analysis. Residual API crystals may serve as seeds for recrystallization, leading to the unstable amorphous state of the formulation [21].

Larger API contents in the sample lead to a decrease in the number of amorphous drugs. Therefore, a complete amorphization could be reached, either by reducing the API content in the formulation, by increasing the processing temperature to slightly elevated temperatures, by further optimizing the screw configuration, or by optimizing the surface properties of the mesoporous silica. Genina et al. demonstrated the melting method using binary systems of ibuprofen or carvedilol with mesoporous silica, for the formation of stable amorphous dispersions with an extruder [9]. However, stability is highly material dependent. For itraconazole in mesoporous silica, recrystallization occurred after 2 months.

To optimize the stability and bioavailability of the amorphous drug, ternary systems have shown good results [6,9]. However, amorphization alone does not account for an improvement in the dissolution of a drug [22]. In addition, the formulation can encounter a dissolution-induced recrystallization of the drug. Therefore, the API dissolution needs to be considered in future studies.

The dry twin-screw processing using mesoporous silica, shown in the current paper, was proven to enable continuous amorphization of itraconazole in the absence of solvents.

## Figures and Tables

**Table 1:** Effect of process parameters on granulate crystallinity.

Process Data						Crystallinity of API			
Screw Config.	T	Throughput	Screw Speed	MRT	*SME*	DSC	DSC	XRD	XRD
	(°C)	(kg/h)	(rpm)	(s)	(kJ/kg)	After 1 Day	After 1 Month	After 2 Months	Crystallite Size (nm)
sc1	20	0.1	30	110	27.1	72%	NA	NA	
sc1	100	0.1	30		54.3	54%	NA	NA	
sc1	200	0.1	30	90	27.1	0%	NA	NA	
sc2	20	0.1	30	200	298.6	26%	NA	NA	
sc2	20	0.3	100	60	150.8	43%	NA	NA	
sc2	175	0.1	30	180	81.4	0%	NA	NA	
sc2	200	0.1	30		27.1	0%	NA	NA	
sc4	25	0.1	100	105	1357.2	NA	2%	44%	13
sc4	25	0.5	250	40	678.6	NA	6%	78%	11
sc4	70	0.5	250	40	1131	NA	0%	28%	4
sc4	175	0.1	100	105	361.9	NA	0%	20%	5
sc4	175	0.5	250	40	113.1	NA	69%	100%	19
Pre-blend								100%	22

## Data Availability

The original contributions presented in the study are included in the article/Supplementary Materials, further inquiries can be directed to the corresponding author.

## Supplementary Materials

The following supporting information can be downloaded at: https://www.mdpi.com/article/10.3390/pharmaceutics16111368/s1, Figure S1: Process set-up consisting of Thermo Scientific^TM^ Pharma 11 twin-screw extruder with open discharge chute (TSG kit) and gravimetric feeder. Figure S2: (A). SEM image of pure itraconazole, (B). SEM image of pre-blend of mesoporous silica and itraconazole; (C). SEM image of granules produced by twin-screw granulation at 25 °C, 100 rpm screw speed and 0.1 kg/h throughput. (D). SEM image of granules produced by twin-screw granulation at 25 °C, 250 rpm screw speed and 0.5 kg/h throughput. Figure S3: XRD patterns of samples of mesoporous silica and itraconazole produced by twin-screw granulation at 70 °C, 250 rpm screw speed and 0.5 kg/h throughput (top), and at 25 °C, 100 rpm screw speed and 0.1 kg/h throughput (bottom). For comparison the XRD pattern of the not processed pre-blend is added (in pink). The green lines indicate the biggest peaks for iron. No iron can be traced in XRD. The peaks in these patterns refer to the API.

## Abbreviations and Nomenclature

API	Active pharmaceutical ingredient
c	Crystalline fraction of API after processing
CPS	Count per second
DSC	Differential scanning calorimetry
HPMC	Hydroxypropyl methylcellulose
$\dot{M}$	Throughput (kg/h)
MPS	Mesoporous silica
MRT	Mean residence time (inside extruder barrel in s)
n	Screw speed (in rpm)
PONKCS	Partial or no crystal structures
PVP	Polyvinyl pyrrolidone
rpm	Rounds per minute
SEM	Scanning electron microscopy
*SME*	Specific mechanical energy (kJ/kg)
TSG	Twin-screw granulation
τ	Extruder torque (Nm)
XRD	X-ray diffraction
ω_i_	Weight fraction itraconazole
