# Supplementary material for: Dry Amorphization of Itraconazole Using Mesoporous Silica and Twin-Screw Technology
Source: Pharmaceutics. 2024 Oct 25;16(11):1368. doi: 10.3390/pharmaceutics16111368 (PMC11597720; doi:10.3390/pharmaceutics16111368)
Supplement: Supplementary file 1 [file pharmaceutics-16-01368-s001.zip › pharmaceutics-3244238-supplementary.pdf]

Supplementary

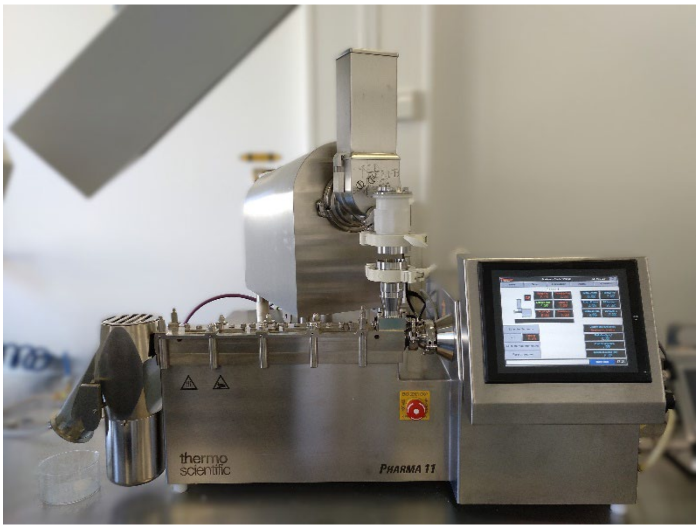

Figure S1. Process set-up consisting of Thermo Scientific™ Pharma 11 twin-screw extruder with open discharge chute (TSG kit) and gravimetric feeder.

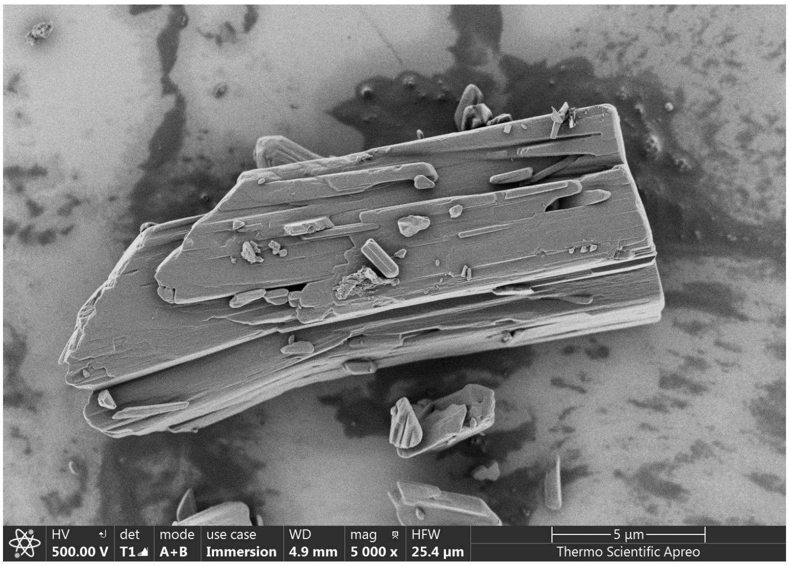

Figure S2(A). SEM image of pure itraconazole,

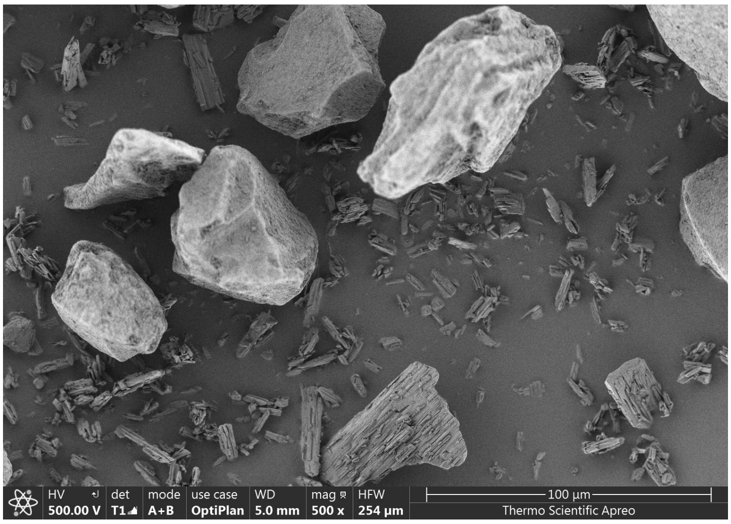

Figure S2(B). SEM image of pre-blend of mesoporous silica and itraconazole.

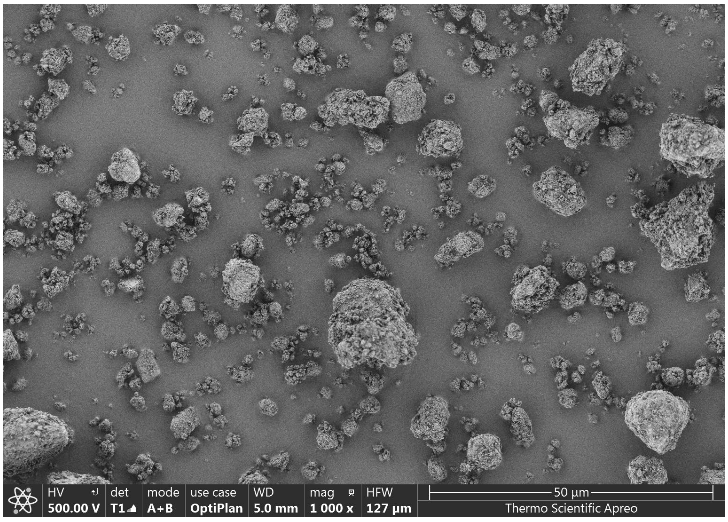

Figure S2(C). SEM image of granules produced by twin-screw granulation at 25°C, 100 rpm screw speed and 0.1 kg/h throughput.

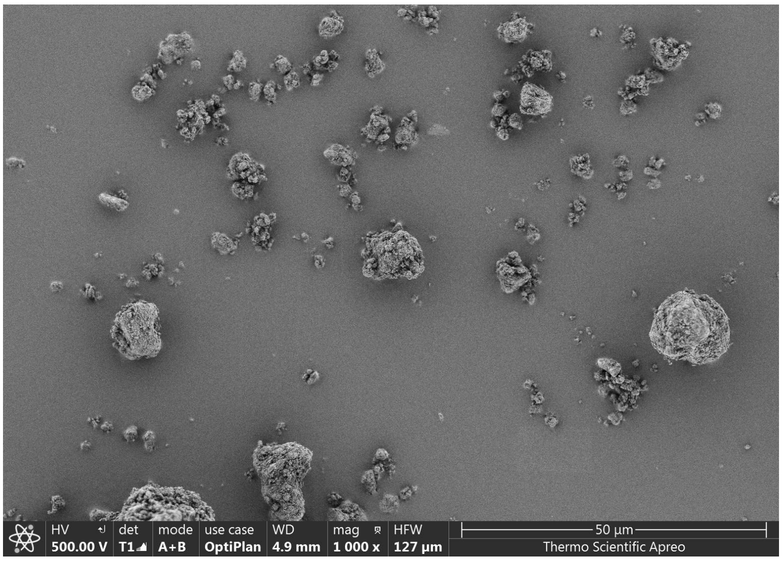

Figure S2(D). SEM image of granules produced by twin-screw granulation at 25°C, 250 rpm screw speed and 0.5 kg/h throughput.

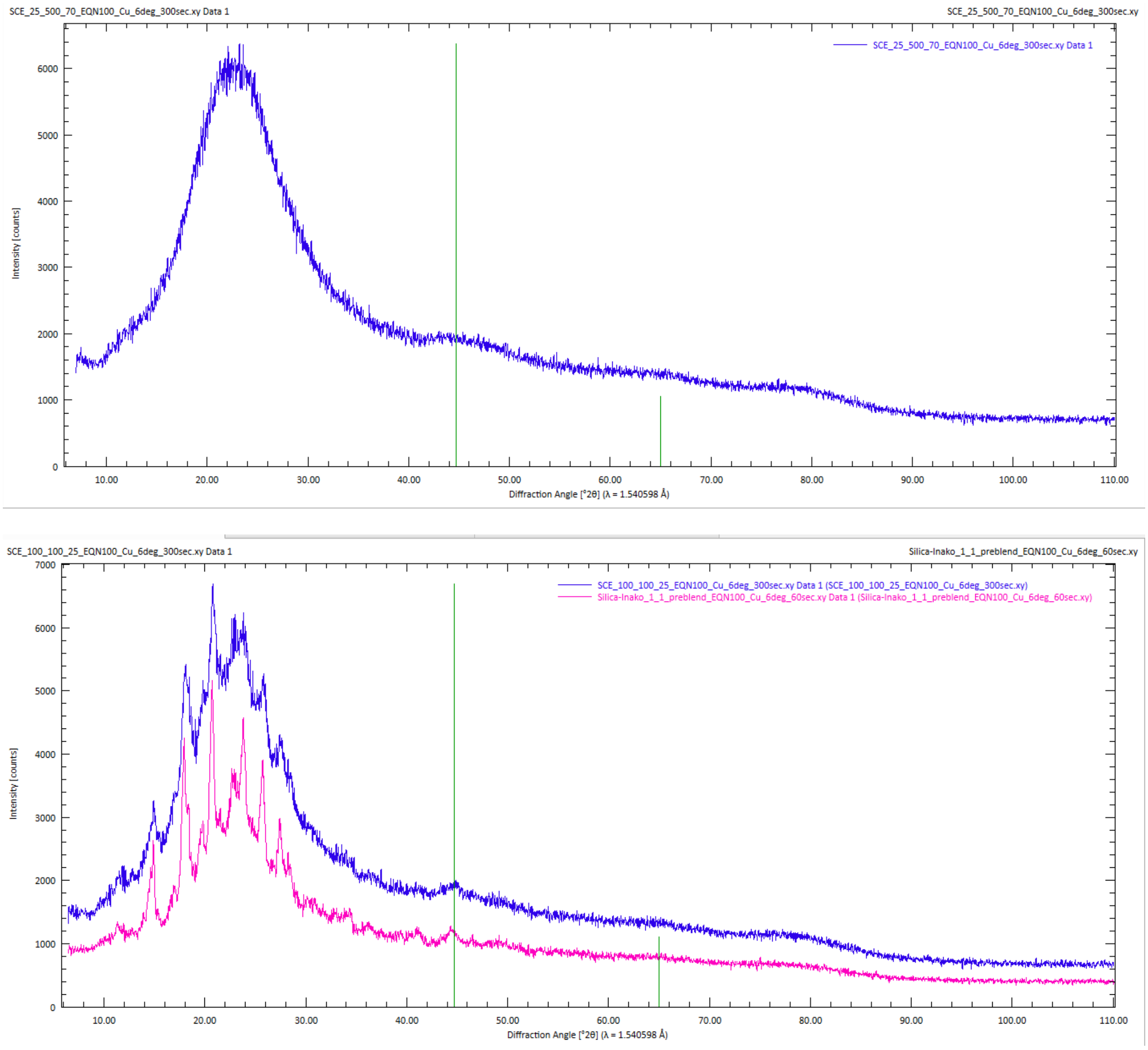

Figure S3: XRD patterns of samples of mesoporous silica and itraconazole produced by twin-screw granulation at 70°C, 250 rpm screw speed and 0.5 kg/h throughput (top), and at 25°C, 100 rpm screw speed and 0.1 kg/h throughput (bottom). For comparison the XRD pattern of the not processed pre-blend is added (in pink). The green lines indicate the biggest peaks for iron. No iron can be traced in XRD. The peaks in these patterns refer to the API.
